# Transactions of the Fourth Annual Meeting of the American Association of Obstetricians and Gynecologists

**Published:** 1892-01

**Authors:** 


### Society Proceedings.

[*From American Journal of Obstetrics.*]

### TRANSACTIONS OF THE FOURTH ANNUAL MEETING OF THE AMERICAN ASSOCIATION OF OBSTETRICIANS AND GYNECOLOGISTS.

HELD IN NEW YORK CITY, SEPTEMBER 17, 18, AND 19, 1891, AT THE ACADEMY OF MEDICINE.

(*Abstract.*)

### FIRST DAY—MORNING SESSION.

THE President, Dr. A. H. WRIGHT, of Toronto, in the chair.

An address of welcome was delivered by Dr. ROBERT T. MORRIS, of New York, and the response was made by Dr. GEORGE H. ROHÉ, Vice-President, of Baltimore.

Dr. AUG. P. CLARKE, of Cambridge, Mass., read an essay on

### POST-PARTUM HEMORRHAGE—ITS ETIOLOGY AND MANAGEMENT.

AFTER an exhaustive and searching review of the conditions leading to the post-partum bleeding, including uterine atony and hourglass contraction, disproportionate growth of the uterine vessels, ectasis of the fundal vessels, uterine edema, placental abnormalities, fibroma or other morbid growths, lacerations of uterus, vagina, or vulva, etc., he discussed the treatment. Anesthesia is of incalculable benefit in lessening many of the dangers incident to parturition, being particularly useful in cases of uterine inertia dependent on the exhaustion of the system generally. In cases of advanced or serious renal affections, chloroform may be safer than ether. Pressure or support over the fundal uterine segments as the child recedes from it will aid in keeping up continuous or regular uterine contraction, and thus lessen the risk of the occurrence of severe hemorrhages. The administration of ergotin or ergotinin will assist in reëstablishing normal contraction. When used hypodermically its physiological and therapeutical action is often speedily and permanently manifested. It is in the milder class of cases that its use will be of the most material service. In cases in which hemorrhage is profuse, intra-uterine injections will be of great advantage. In women of full or plethoric habit, cold water may be employed ; in those who suf fer from nervous affections water from 115° F. to 125° F. is to be preferred. Doubtless, when hemorrhage is arrested by hot water it is owing to the formation of thrombi more or less extended into the vascular tissues. The employment of cold has a reflex action,—it gives a toning effect to all the tissues, it facilitates the constriction of the muscular coat of the dilated vessels. Caffeine, used hypodermically, is of benefit. The application of some form of electricity will aid in some measure in promoting contraction of the uterine muscular fibres. In cases in which hemorrhage is anticipated, the early administration of quinine may in large measure serve to keep the hemorrhage under control. The occurrence of certain pains may lead us to anticipate post-partum hemorrhage. If the pains are acute and brisk, with abrupt endings, and followed by unusually long pauses, we may infer that there is a deficiency of nerve force. This may result in atony of the muscular structures and in failure to effect constriction of the uterus and closure of the utero-placental vessels. In cases in which hemorrhage proceeds from the lower section of the uterus or from the upper portion of the cervix, the application of iodoform wool and gauze, and of styptics or of iodine, will be of service. The author has great confidence in the employment of nitrite of amyl ; it is an arterial and cardiac stimulant of the most extraordinary power. The employment of intravenous injections, and the dangers attending their use, were matters for determination in each individual case. The employment of alcoholic saline intravenous injections for their dynamic or tension effect will be most beneficial. The author's later experience favors the adoption of the method of hypodermic injection or transfusion of spirituous saline solutions. This method is more convenient, is safer, and is more likely to be followed with favorable results. Other methods for controlling hemorrhage and for preventing collapse are referred to. Compression of the abdominal aorta should sometimes be tried. This may, in some measure, enable the medical attendant to get control over the hemorrhage when all other means have failed. This procedure has been approved by such great authorities as Barnes, Churchill, and Simpson.

Dr. Robert T. Morris, of New York, read a paper on

### A NEW METHOD OF PALPATING THE KIDNEY.

Dr. W. J. Asdale, of Pittsburg, read a paper on

### REMOVAL OF THE KIDNEY IN DISEASE, WITH CASES.

Before reliable conclusions and methods can be established, and this most formidable surgical procedure can be made safe, if ever, a familiar acquaintance with all that has been done in the sphere of renal surgery must be secured and the details of a considerable number of cases must be carefully studied, that questions of both physiological and pathological import may be determined. Any contribution, although stating no new facts, may be of present value by corroboration and emphasis of points previously taken.

The following points were accentuated by the histories of a number of cases and operations:

First, in respect to the symptomatology of malignant disease. It is often insidious in its attack; pain may be absent or insignificant in amount. Early copious hemorrhages, without any marked previous manifestations of concern, are most suggestive of structural change of malign character.

Second, in regard to method of operation. The choice will be governed not more by preference than by necessity. We are reminded, however, of the facility of approach by the lateral abdominal method of incision, and of the ease with which large solid growths may be taken from the renal site. Again, in all cases it is of primal importance to possess the advantage of direct palpation of the other kidney before nephrectomy; this manœuver the operation by primary anterior incision makes easy.

The antero-lateral incision provides the minimum of injury to the peritoneum and the strongest assurance that soiling of the peritoneal surface will be avoided. Drainage, the importance of which cannot be overestimated, can be most efficiently applied after the lateral abdominal operation. Shock, even to the aged and feeble, does not of necessity inure to forbid a carefully conducted operation for removal of the kidney. Finally, the importance of early diagnosis and the futility of late operations, in malignant disease especially, are clear.

DISCUSSION OF THE TWO PRECEDING PAPERS.

Dr. A. Vander Veer, of Albany: We owe Dr. Asdale our thanks and gratitude for his thorough and candid manner of reporting his cases; there was a fatality present that no operator can avoid—that is, an advanced condition of disease. Malignant disease of itself is always a dangerous condition for us to attack, and when advanced, as in these two cases, we have heavy odds against us. The consensus of opinion is that chloroform is the safest anesthetic to use in surgical kidney. In malignant disease let us operate early,—as early as possible.

Dr. C. A. L. Reed, of Cincinnati: The question of the treatment of the pedicle in nephrectomy will remain a serious one as long as we have kidneys to remove. We all recognize the treach, erous friability of the renal vein. To avoid the unhappy and almost necessarily fatal accident of cutting it by a tight ligature-he had put around this vessel the protecting influence of its neighboring structures, and had ligated the pedicle in one mass. He endeavored, in effecting the division, to leave something of a button to make the ligature secure. So far he had not been embarrassed with secondary hemorrhage. The amount of force that is required to control hemorrhage from the renal artery need not be so great as to cut the walls of the neighboring vein, providing we have left a sufficient button to prevent slipping of the pedicle under the very considerable circulatory pressure which is brought to bear upon it, during the few hours immediately succeeding the operation. Unless there be surgical conditions of the ureter itself demanding special treatment, there is no need of treating it other wise than you would the circulatory vessels.

Dr. Kellogg, of Battle Creek, Mich.: The question which should be raised in this discussion is, whether it is better to remove the kidney, or whether it is better to perform the operation of nephrotomy. If we drain the kidney in case of suppurating kidney and in case of malignant disease of the kidney, the patient will likely recover. Mr. Tait never removes the kidney. If any operation at all was considered desirable, he performed the operation of nephrotomy through the lumbar region and drained the kidney. K. made it a practice to examine the position of the kidneys and all abdominal organs in every case of pelvic disease of women. In a very large proportion of cases the right kidney especially is prolapsed and movable. In thirty per cent. of all cases in which there is displacement of the pelvic organs, there is also displacement of the kidneys. His method has been to first examine the patient on the back, the shoulders elevated, and legs flexed forward so as to relax the abdominal muscles as much as possible; placing one hand at the back and the other hand in front. In case he fails, he has the patient rise on the feet and rest against the end of the table; then, on bending forward, the abdominal muscles are completely relaxed, the kidney is dragged down, and when the patient takes a deep breath it is easy to seize it, if it is at all prolapsed.

Dr. J. H. Carstens, of Detroit: I am much interested in the diagnostic points made. In all diseases of the kidney there is great danger in using ether. In all such cases it is advisable to use chloroform. Looking back upon cases which have died within twenty-four to forty-eight hours, reported dead from shock, I am confident now that they died from the ether. Even chloroform will produce congestion of the kidney, but not to the extent ether does.

Dr. Henry T. Machell, of Toronto, asked for points which would enable us to recognize the kidney after the abdomen is opened. He had seen in more than one instance a considerable loss of time in recognizing and determining what was or what was not the kidney.

Dr. Morris, of New York, said that as to the question of how we can determine whether we have kidney or some other organ, he had only once been in the position where he could not tell from the character of the capsule and the tissue of the organ whether he had kidney, liver, some morbid growth, or some other organ. In that case he had colloid carcinoma; he found the aorta, then the renal artery and traced that, and determined from the relative position of the renal artery that the kidney was beneath this mass.

Dr. L. S. McMurtry, of Louisville, read a paper on

### INTRAUTERINE IRRIGATION AFTER LABOR.

The author referred to the relation of microörganisms to septic infection during the puerperal state, and insisted upon the application of aseptic methods in the lying-in room. He also referred to the fact that even modern treatises on midwifery were inexplicit in giving directions with reference to the essential methods of prophylaxis, and that they failed to lay down definite lines of treatment for the initial stage of puerperal sepsis. He further insisted that these cases should be dealt with promptly at the very outset by systematic employment of intra-uterine irrigation after labor, which should be done with all the care of an important surgical procedure. He also stated that the danger which had been attributed to this method was not a real one, if the lines laid down for its employment were carried out with exactitude. He insisted that the operation should be performed by the surgeon in person, and that antiseptic agents were not essential, provided an absolute cleanliness was maintained, and that irrigation was carried on to the extent of removing all the débris and decomposing material which lodged in the uterine cavity.

The author employs for this purpose a fountain syringe, provided with a glass nozzle, and loaded with clean water, that has been previously boiled. It must be used quite warm and the patient's clothing must be drawn out of the way, while the extremities are to be protected with blankets. A free exit for the return flow must be provided. When this is done in a careful way with water at the proper temperature, the patient's comfort is enhanced, and her improvement, as a rule, is immediate. The principle of treatment is that of flushing and drainage, the efficiency of which has been demonstrated in similar conditions in pelvic surgery.

Dr. W. W. Potter, of Buffalo: The distinguished Fellow from Kentucky sounded the keynote of this whole question of intra-uterine irrigation after labor, when he said that the time for commencement of the treatment was at the time when it became essential—that is, at the initial symptoms of infection. If we could only always determine when the initial symptoms presented, I have no doubt that this method would result in the saving of life and in the prevention of prolonged sickness. There are ways in which infection gets to the vital organism insidiously, which we only recognize by watching the symptoms which it produces; hence the importance attaching to the careful watching of the puerperal woman by her attendant,—not treating a labor case lightly, not going to it and hurrying away in a few minutes after delivery, and saying "I will come back when you need me,"— but she is to be watched with all care, even after simple labor, for a few days, until all danger of the infective invasion has passed away. It is important to emphasize all this, for the obstetricians of today must certainly recognize the fact that they are occupying a more responsible place than ever. There is more light upon the subject than formerly.

Dr. E. E. Montgomery, of Philadelphia: This subject is one of vital interest, for upon the meeting and subduing of the germs at an early stage is dependent the future comfort, health, and possibly life of the individual. He fully indorsed what was said as to the importance of early intrauterine irrigation where there is the least indication of septic infection. We have in the cavity of the uterus a large absorbing surface; a surface that is covered with débris, a surface in which, through the heat of the body and the character of the secretions, germs multiply with great rapidity, are readily taken up and carried through the vessels, carried by the continuous action of the mucous membrane into the tubes, and we have secondary infection not only of tubes and ovaries, but we have systemic infection through the absorption into the system. It is important to render this surface sterile early and prevent the development of the disease. In such cases he would advocate, in addition to irrigation, the use of the curette, the scraping away and removal of the infected débris, and, after irrigation with a chemical solution, the introduction of a twist of gauze to the fundus, and in this way make sure that the subsequent drainage was perfect and complete.

Dr. Geo. H. Rohé, of Baltimore: It is my conviction, based upon observation and some personal experience, that the practitioner who is in doubt about antisepticism in obstetrics will lose nearly as many patients from septic troubles as one who disbelieves in that method. If there is any one thing necessary in practising aseptic obstetrics, it is a firm belief that it is absolutely necessary in every case. Consequently it has been well said that the time to begin treating sepsis in a lying-in woman is before she is septic. But even after the septic condition has been established, a thorough carrying out of the antiseptic practice will result in success in a large majority of cases. Any one who has ever seen the interior of the uterus of a woman who has died of septic infection after delivery, will appreciate the importance of more than superficial measures,—not merely an injection now and then, even thoroughly made, but also the use of some chemical disinfectant which will inhibit the rapid multiplication of the germs.

Dr. J. H. Carstens, of Detroit: It has been pretty well settled that normal cases had better be let alone; but where symptoms develop, it is well to start irrigation very early. There are cases where the temperature rises up to 103°, or 104°, or 105°, where the irrigation has no effect at all, even if you irrigate every three hours, or two hours, or every hour. There is no débris there; nothing wrong with the uterus; the physician or midwife who attended the wound was aseptic, and still that woman has puerperal fever. These are cases of auto-infection. We know that when women have a latent disease of the tubes, be it tubercular, gonorrheal, or an ordinary pyosalpinx, the act of parturition will cause it to break out in full force or will cause a rupture of the tube, which will allow pus to run down into the uterus and there set up a violent sepsis. These are the cases which need laparatomy. We ought to have it before our minds that there are cases which are due to a poison being introduced from without, by the physician or nurse, and there are other cases where the cause is within the patient and may have been lying latent for years, simply needing something to cause the explosion.

Dr. Cushing, of Boston, in confirmation of what the last speaker said, reported a case that apparently sprang from tubal infection.

Dr. A. H. Wright, of Toronto: I indorse the statements expressed in the paper. The subject is of the utmost importance. Nothing in the art of obstetrics has given me more anxious thought than this question of antisepsis. It is my practice in the lying-in hospital and in private practice to use intrauterine irrigation very seldom. In itself it is an evil, capable of doing a certain amount of harm. When the necessity arises I certainly do not scruple at once to go on with irrigation in the interior of the uterus. As far as I have seen irrigation carried on by general practitioners, I have been sometimes rather horrified at the miserably careless and indifferent way in which it was done. It is a very difficult thing to teach hospital students how to do this properly.

Dr. J. F. W. Ross, of Toronto: I do not think ordinary water used as an injection is as good as some antiseptic solution. My experience with intrauterine irrigation has not been as favorable as I could wish. Two cases of puerperal septic trouble coming under my notice within the last two years have been treated by packing the uterine cavity with iodoform gauze through a speculum, and in this way attempting to subdue the formation of the poisonous ptomaines in the cavity.

Dr. Kellogg, of Battle Creek, said there was another use or irrigation which had not been mentioned. In a case in which the temperature rose to 104½°, irrigation was employed, but did no good. By the application of a hot douche, 140°, the uterus was made to contract. The next morning the temperature was normal, and did not rise again. His plan of using the douche is to introduce a large drainage-tube, then a small catheter through the drainage tube, and then to use water at a temperature of 130°. Lower temperature is often the reason for failure. Warm water relaxes, hot water contracts. Very hot water is efficient as a germicide. The uterus will bear a still higher temperature.

Dr. McMurtry, closing the discussion: I feel very grateful to the Fellows for the very cordial manner in which they have received the suggestions I intended to convey. The purpose of the paper was not to discuss the routine use of intrauterine irrigation after labor, or to deal with the prophylaxis of puerperal sepsis, but simply to emphasize the point that this very valuable method, which we can institute in the very initial stages of sepsis, is not generally appreciated by the great body of the profession; that the golden moment when it can be most efficient is lost by the administration of a hypodermic dose of morphia, or other tentative measures, under the mistaken idea that the initial stage of sepsis is a little milk fever or malaria, or some little disturbance brought on by the process of labor. Dr. Carstens, of Detroit, has alluded to a class of cases which should not be considered in connection with this treatment at all—that is, to the fulminating cases, cases of sapremia, where in a few hours the system is thoroughly saturated with the poison; cases that nothing in the world can resist. Even in the initial stages of these cases this treatment can do no harm. The cases alluded to by Dr. Cushing are scarcely within the scope of the discussion. There is no such thing as auto-infection of a woman after labor. Cases of tubal disease belong to that class where the disease was present before labor began. They may have been mechanically affected by the process of labor and the muscular contractions, so as to complicate the case. They are complications of the puerperal condition. Moreover, the treatment of those cases by laparatomy, evacuation, removal of the disintegrating structures, drainage, and irrigation, is but an application from above of the same principle of treatment.

### First Day—Afternoon Session.

Dr. Llewellyn Eliot, of Washington, D. C., read a paper,

### IS A CHILD VIABLE AT SIX AND A HALF MONTHS?

He referred to the French law, which excludes the possibility of the viability of a child born before the sixth month (one hundred and eighty days), as unjust, since cases have occurred where children born before that time have been reared and lived for many years. He denied the plea of superfetation, in these cases, as untenable. A table comprising cases in which the period of utero-gestation extended from the fourth month, (one hundred and twenty days) to the termination of the seventh month, supplements the paper. Dr. Eliot related the histories of three cases of early viability, one at six months and eleven days, one at seven months and one day, and one at seven and a half months, and drew the following conclusions: 1. A child under peculiar circumstances of development is viable at four months. 2. A child is viable at six and a half months. 3. The moral character of the parents has nothing to do with the birth of a premature child, when considered from a standpoint of constitutional development. 4. Obstetricians should strive to convince jurists of these facts.

### DISCUSSION.

Dr. H. O. Marcy, of Boston, reported the case of a syphilitic woman with a healthy husband. Both desired children, but she had aborted eight times. He had attended her two or three times in abortion at the fourth or fifth month. In her last pregnancy at about the fourth month she had slight indications of abortion. At about the sixth month she aborted. The fetus was living and was at once placed in an incubator. It is now a strong young boy.

Dr. J. H. Carstens, of Detroit: The paper of Dr. Eliot is one of great importance from a medico-legal standpoint. I would hesitate to say that a child was five and a half or six months, or six and a half months. I do not see how it is possible for us to say how long a child has been in utero. A woman may have a discharge of blood similar to menstruation when she is already pregnant for a month. In the present state of our knowledge it is clearly impossible to say how old that child is, unless you have two absolute factors: that you have the woman menstruate at a certain date, and that coition was had only at one certain date. You cannot even judge from the time the woman feels life, because that varies.

Dr. Eliot, of Washington, D. C., closing the discussion, said that in using the incubator it was necessary to regulate the amount of moisture as well as heat. If we have it too dry, we kill the child; if we have it too hot, we kill the child.

Dr. E. E. Montgomery, of Philadelphia, read a paper on

THE APPLICATION OF SACRAL RESECTION TO GYNECOLOGICAL WORK, in which he advocated the procedure in all cases in which uterus and rectum were both involved with malignant disease, and in cases of uterine cancer, where the uterus was enlarged or where the vagina was small and the case complicated by disease of tubes and ovaries, causing extensive adhesions.

He places the patient upon the left side or semi-prone position, and makes a bow-shaped incision from the right sacro-iliac synchrondrosis across the median line to a little beyond the apex of the coccyx, enucleates the latter bone, separates ligaments and muscles from the right side of the sacrum, and, beginning just below the third posterior sacral foramen, cuts off with chain saw or bone-pliers the right ala of the sacrum.

In operations for removal of the uterus and its appendages, the rectum is pushed to the left and the peritoneum opened. This brings the operator upon the posterior surface of the uterus, when the broad ligaments may be seized by hemostats, raised up, the broad ligaments ligated, and the uterus removed. After removal of the organ, the peritoneal surfaces may be stitched over the vagina and the posterior peritoneal opening also closed. He does not prefer it to vaginal hysterectomy where conditions are favorable for the latter. He reported two cases in which he had done the operation; one for cancer of the rectum and uterus, in which three inches of the rectum and the uterus and appendages were removed and the calibre of the gut restored. A large collection of feces pushed up the lower segment of the rectum, requiring the wound to be reopened and a secondary operation four weeks later. The second operation was done for cancer of the uterus, complicated by tubal and ovarian disease with adhesions. Both patients recovered, and no inconvenience in locomotion was experienced.

Dr. C. A. L. Reed, of Cincinnati: This operation attracted my attention when the first publication of it appeared. Like many of the other operations, particularly those that involve the invasion of structures that we have not been in the habit of treating surgically, it appears to be more formidable than perhaps it really is. In an effort to treat malignant disease involving the middle segment of the rectum, this operation would be demanded and would be justifiable, for we are justified, perhaps, in doing almost anything for the relief of malignant cases, particularly those involving important tissues, such as the rectum and uterus; but if we can bring the maximum of relief with the minimum of risk, that is the line that we ought to follow. There is one question which cannot be answered as yet from any ascertained results, and that is with reference to the remote influence of this operation. The removal of the coccyx and the removal of the lower segments of the sacrum, must of necessity deprive the lower portion of the pelvis of an important basis of support; and what is the condition of our patients with regard to the support of the superimposed viscera following the operation, after a considerable length of time? Dr. Montgomery's cases are yet too recent to afford an answer to this question. While the primary results have been very good, it would have been vastly better to have relieved his patient by primary colotomy; but if this operation will bring the same amount of relief with as little risk of primary mortality, and at the same time insure the patient voluntary control of her fecal discharge, by all manner of means let us encourage it.

Dr. H. O. Marcy, of Boston: We ought to lay emphasis upon primary colotomy. I mention it simply because I lost two patients where the result might have been entirely different if I had done colotomy first. This was in cancer of the rectum. When we recollect that the intestine is very fully distended with gases and feces, the pressure upon our sutures is something enormous. Primary colotomy gives us that all-important factor of surgical rest of the tissues with a far better promise of success.

Dr. H. T. Hanks, of New York, thought that this operation could be recommended in most cases of chronic pelvic abscess where a rupture has taken place into either the vagina or rectum, and where the tissue underneath the broad ligament is honeycombed. He was interested from the fact that he had had one or two cases where he probably would have succeeded better by doing this operation. It is a difficult matter to cut through the abdominal wall into the true pelvis and find out the exact condition. Though we very quickly get a view of the parts, we cannot manipulate easily.

Dr. W. H. Wathen, of Louisville, congratulated Dr. Montgomery upon his courage, his tact, and his excellent technique.

Dr. A. Vander Veer, of Albany: I would not be willing to abandon vaginal hysterectomy for removal of the uterus and ovaries. The technique of vaginal hysterectomy is so perfect, it is one of the most brilliant operations we have to perform at the present time, and the results are satisfactory. In regard to colotomy, I never had a patient who was thoroughly satisfied with the result of the operation. All were dissatisfied with the fecal discharge. But in the five or six cases in which I have operated for removal of the lower segment of the rectum, it mattered not if there was some leakage, some trouble in keeping a pad there and receiving the feces; they all say, "Doctor, it comes out at the right place. It feels more natural."

Dr. Ap Morgan Vance, of Louisville: The performance of primary colotomy would bring about a difficulty from the fact that a good deal of the gut was to be removed; and if there is cancer there, the more removed the better. If it was tied, anchored, at the point of ordinary colotomy, we would have difficulty in bringing it down to get approximation.

Dr. J. F. W. Ross, of Toronto, read a paper entitled

### HOW SHOULD WE PROCEED WHEN ABDOMINAL TUMORS ARE COMPLICATED BY PREGNANCY?

He emphasized the point that there was nothing of malpractice in the opening of an abdomen during the existence of a concealed pregnancy, before proceeding to discuss cases in which pregnancy has been recognized. Cases of ovarian tumor and fibroid tumor of the uterus were reported, and a request was made for reports from members of the association, so that a foundation might be laid on which to build up a few fixed rules for future guidance. Ovarian and myomatous tumors were the only two forms taken into consideration.

*Ovarian Tumors.*—He said that the methods of treatment to be discussed were :

1. To allow the pregnancy to go to full term, or until the uterus throws off its product.
2. Puncture of the cyst until delivery is completed.
3. Induction of premature labor.
4. Ovariotomy—the uterus left to abort or go to term.
5. Ovariotomy—the uterus emptied of its contents by incision.
6. Ovariotomy and abdominal hysterectomy.

The author advocated early ovariotomy, but supported cyst puncture in certain favorable cases, if the patients objected to other operation or wished to have a living child. If at any time bad symptoms arose, he insisted on immediate abdominal section. In advanced cases, where injury or much handling of the uterus is unavoidable, the organ should be emptied to forestall the almost inevitable abortion or premature labor.

*Myomatous Tumors.*—1. Induction of premature labor.

2. Early myomotomy or abdominal hysterectomy.
3. Late hysterectomy or Cesarean section.
4. Tentative measures, as :
   (*a*) Enucleation of cervical tumor to permit labor completion.
   (*b*) Enucleation of a sloughing tumor following labor.
   (*c*) Abdominal hysterectomy for a sloughing tumor or uncontrollable hemorrhage following labor.
   (*d*) Abdominal hysterectomy for septic infection from retention of discharges in the non-contractile uterus.
   (*e*) Abdominal hysterectomy or Cesarean section to end a labor that will require long forceps, version, or craniotomy.

He finally concluded that the tentative measures were the best.

Dr. H. T. Hanks, of New York : I have been interested for many years in the subject of uterine and ovarian tumors. We not only want to consider the patient but the surroundings, and when you know that you have got a unilocular cyst, that you can tap and remove the fluid and the patient can go on to term, or at least to eight and a half months, you are justified in doing it. But if the pregnancy is complicated with fibroid tumors, the case is different. They grow very rapidly from the first month up to the fifth or sixth month, but do not from the seventh to the ninth month. If the tumor is situated in the cervix you should enucleate it, because you cannot deliver through a cervix of which two-thirds is a fibroid. If you cannot do that, you are justified in producing premature labor or an abortion at the second or third month. If the tumor is the size of your fist, and you can push the cervix above the brim, and you have two-thirds of the cervical tissue healthy, you are justified in delaying. If the tumor is above the middle zone the child can be delivered quite easily at term.

Dr. A. Vander Veer, of Albany: The difficulty of diagnosis in a case of fibroid of the uterus or ovarian tumor is one of the problems of surgery. The subject is now being handled with much greater clearness and more satisfaction, but it is essential to make a diagnosis, and in making the diagnosis we have very little that helps us in the history given by the patient. A condition which Dr. Ross did not touch upon is this, that in most cases where a patient who has a uterine fibroid becomes pregnant, the tumor will take on a certain amount of growth,—more in some cases than others. Occasionally it undergoes a sarcomatous change. Again, a patient may have a fibroid, go through pregnancy and a safe delivery, after which the fibroid will disappear. The medico-legal point of this question has been touched upon by two or three of the decisions that have occurred in court. We should be thoroughly united and thorough in our emphasis that in these cases the fibroid does sometimes disappear under the influence of pregnancy. In the treatment of ovarian tumors coincident with late pregnancy, we should tap and carry the patient along as near as possible to the full time.

Dr. I. H. Cameron, of Toronto: I am strongly in accord with the opinions expressed that no general rules can be laid down for our guidance in any case. Every case, as Dr. Hanks has said, must be treated on its own merits. A sharp distinction should be made between tumors involving the uterus and those involving the ovaries. As has been said, the position of fibroid tumor makes all the difference in the world. If it be clear that from the position of the tumor it will not interfere with delivery, it may be laid down as a general rule that it should not be touched before gestation is complete. Of course, there may be risks of inflammation or malignant disintegration. I would like to enter my protest against tapping a unilocular cyst as a palliative measure. Twice in the last week I have had occasion to witness the very great danger of tapping.

Dr. H. O. Marcy, of Boston, said that he agreed with Dr. Cameron. He supposed tapping under these circumstances was out of the question. He reported a case showing its danger.

Dr. W. W. Potter, of Buffalo, reported a case of successful operation for removal of a tumor during pregnancy.

Dr. H. O. Marcy, of Boston, asked what was the condition of the veins.

Dr. Potter replied that they were highly congested. He believed that it was a case where tapping would have been very bad practice. His experience and that of others led him to believe that tapping should never be employed ; if anything was done, it should be through an incision.

Dr. J. H. Carstens, of Detroit : I am inclined to think that because of the peculiar blood changes, union takes place better during pregnancy than at any other time. I wish emphatically to protest against the tapping of tumors during pregnancy. Cases of ovarian tumor, no matter what kind they might be, occurring during pregnancy, ought to be operated upon. In case of fibroid, you have to judge of each individual case yourself.

Dr. A. Vander Veer, of Albany, said he did not suppose there was any man in the State of New York who had in his teachings emphasized more this point in reference to tapping in the case of an ovarian tumor than he.

[*To be continued.*]

---

Dr. Philip S. Wales, U. S. N., Medical Director National Museum of Hygiene of Washington, recommended to Lieut. Perry, the Arctic navigator, that the underwear of the exploring party should be so constructed as to prevent radiation, and also encircle the body with a stationary atmosphere. The theories for clothing of Profs. Von Petten Koffer, Director Hygienic Institute, Munich, and Parkes of London, have also a bearing on the above and they are conceded to be embodied in the Jaros Hygienic Underwear. The excellent results with an extra heavy fabric of these garments in the United States Army North Posts, have made it possible to successfully withstand a temperature of forty-seven degrees below zero and in a thirty mile ride without buffalo coat.—*The Prescription.*

